# Study on Proportioning Scheme of Coal System Rocky Similar Material Based on Orthogonal Test

**DOI:** 10.3390/ma16227113

**Published:** 2023-11-10

**Authors:** Lin Sun, Wan-Xu Wang, Ji-Shan Xu

**Affiliations:** School of Resources and Geosciences, China University of Mining and Technology, Xuzhou 221116, China; ts22010144p31@cumt.edu.cn (L.S.); wwx17860507167@163.com (W.-X.W.)

**Keywords:** similar geotechnical materials, similar models, orthogonal experimental design, material proportioning, physical modeling tests

## Abstract

Similar materials play an important role in model testing. In order to meet the demand for similar materials in modeling tests, such as those on coal mining, coal system rocky similar materials were formulated using yellow sand as a coarse aggregate, heavy calcium carbonate as a fine aggregate, and cement and gypsum as binders. Based on the orthogonal experimental design method, four influencing factors, namely the aggregate–binder ratio, heavy calcium carbonate content, cement–gypsum ratio, and moisture content, were selected. Each factor was designed at five levels. Through weighing, uniaxial compression, Brazilian splitting, and variable-angle plate shear tests on 225 specimens under 25 different ratios, five physico-mechanical property indicators of the material, including density, compressive strength, tensile strength, cohesion, and internal friction angle, were obtained under different ratios. The test results indicate that the similar materials formulated with the above raw materials had a wide range of mechanical properties, which met the simulation needs of different types of coal rocks, such as main coking coal, anthracite, shale, etc., in the similar model test. Range analysis was adopted to analyze the sensitivities to each factor, which showed that the density and internal friction angle of similar materials are mainly controlled by the aggregate–binder ratio; the cement–gypsum ratio mainly controls the compressive strength, tensile strength, and cohesion of the material. Analysis of variance (ANOVA) was adopted to analyze the sensitivities to each factor, which showed that the aggregate–binder ratio had a highly significant effect on the density of the material, the cement–gypsum ratio had a highly significant effect on the compressive and tensile strength of the material, the cement–gypsum ratio had a significant effect on the cohesion and density of the material, and the moisture content had a significant effect on the compressive strength of the material. The remaining factors did not significantly affect the material parameters. The results of this study can provide some reference for the selection of coal system rocky similar materials in subsequent physical modeling tests.

## 1. Introduction

Underground coal mining reduces the strength of the original strata and deformation damage occurs [1]. This not only poses a potential threat to safety, but also may cause other geological disasters. As coal mining is a large-scale underground project, it is difficult to accurately predict and estimate the damage at the actual site. Therefore, current research often utilizes similar model tests to simulate the coal mining process. As an important means of research in the field of geotechnical and geological engineering, the similar material modeling test method has been widely used in engineering simulation studies related to geotechnical bodies, such as coal seam mining [2,3,4], foundation pit excavation [5,6], slope management [7,8], tunnel boring [9,10], and so on. As a basic component of the similar model, the similar material is an artificially prepared material used to simulate a geotechnical body under real working conditions. It should have the same structure and some or all of the physical and mechanical properties as the target geotechnical body. In similar simulation tests, whether the physical and mechanical properties of similar materials are consistent with the actual engineering situation directly determines the accuracy of the simulation and the success or failure of the test, so the selection of suitable similar materials is crucial for similar material simulation tests.

In order to obtain simulated materials suitable for different engineering conditions, scholars at home and abroad have conducted a lot of research on the formulation of similar materials and achieved fruitful results. In the 1960s, Italian and Portuguese scientists conducted many small-scale modeling experiments with mixtures of lead oxides (PbO or Pb_3_O_4_) as the main material [11,12]; Han et al. formulated a new geomechanical modelling material (MIB) geomechanical modeling materials with high bulk density, low elastic modulus, and low strength, and improved them to expand the scope of application [13,14]; a high-capacity geo-mechanical model material (NIOS) model material was blended by Ma et al. [15]; Wang et al. developed an iron crystal sand cementitious material (IBSCM) with high bulk density and low elastic modulus [16]; Li et al. developed low- and medium-strength coupled flow-solid similar materials (SCVO) that can simulate different permeabilities through extensive proportioning tests [17]; Fu et al. developed a similar material for pulverized sandy mudstone with water-absorbing and swelling properties based on the physical and mechanical properties of pulverized sandy mudstone [18]; Zhao et al. formulated class concrete materials for tunnel lining [19]; Zuo et al. successfully modeled limestone rock medium with quartz sand, gypsum, and cement [20]; Zhang et al. [21] and Wang et al. [22] investigated salt rock for oil and gas storage media and similar materials used for creeping soft rock, respectively; Diao et al. formulated a similar material to simulate swelling rocks [23]; Wen et al. proposed a new artificial mudstone similar material with an adjustable component ratio [24]; finally, Yang et al. reported a scheme for the preparation of similar materials for conglomerates under osmotic stress coupling [25]. In terms of simulation materials for coal seam mining, Li et al. formulated a low-strength analogous material with sand, calcium carbonate, and gypsum to meet the mechanical properties of coal rock [26]; Wang formulated coal-like materials based on the homogeneous design method utilizing cement, sand, water, activated carbon, blowing agent, crushed coal, and crushed coal as the main materials [27]; Yang et al. formulated a coal bed floor sandstone similar material using sand, calcium carbonate, and gypsum as raw materials [28]; Dai et al. successfully modeled coal with cement, sand, water, activated carbon and coal dust [29]; Liu et al. formulated coal rock similar materials using cement, sand, nitrile rubber powder and polystyrene [30]; and Miao et al. studied the height of fractured water-conducting zone in undermined rock strata in underground coal mining using similar materials [4].

Upon summarizing the previous research efforts, it is evident that there are the following problems in the current research: (1) the research results for coal simulation materials are single, and there is a lack of systematic development of similar materials for coal rock; (2) most of the current research focuses on the change rule of the physico-mechanical properties of the materials with the proportioning scheme and does not specifically indicate the kind of rock that the developed similar materials can simulate; (3) even if simulating the same kind of raw materials, different scholars have chosen different kinds of raw materials and there is a lack of uniform standards, which has led to the uneven nature of the developed materials. Even if, in the simulation of the same kind of raw rock, different scholars choose different kinds of raw materials, there is a lack of uniform standards, leading to the properties of the materials not being uniform. In addition, scholars have not reached a consensus on the amount of water that should be used for mixing, and few have considered the effect of moisture content on the strength of similar materials.

In response to the aforementioned issues, this study comprehensively investigated the proportioning scheme for coal system rocky similar materials through an orthogonal experimental design. The primary objective was to create a coal system rocky similar material suitable for a wide range of simulation purposes. In accordance with previous research, the four most commonly used building materials, including cement, gypsum, yellow sand, and heavy calcium carbonate, were chosen as raw materials. A series of experiments were conducted utilizing tap water as the mixing medium without any additional additives. The outcomes of this study can serve as a valuable reference for indoor modeling tests related to coal seam mining and physical modeling tests related to coal strata. It is anticipated that these findings will offer clearer guidance for the development and utilization of similar materials in the context of coal rock.

## 2. Similar Material Proportioning Program Design

### 2.1. Selection of Raw Materials for Similar Materials

Generally, simulated materials need to simulate the structure and properties of rocks. The structure of rocks is commonly modeled using sandy and chalky materials, and the mechanical properties are commonly modeled using cementitious materials. In addition, a great deal of experience has shown that the selection of similar materials needs to fulfill the following conditions:

(1) A wide range of variation in mechanical parameters of similar materials allows the simulation of different types of rock materials;
(2) The material does not rust and has high insulating properties;
(3) Inexpensive and readily available materials that can significantly reduce test costs;
(4) The material is non-toxic and does not cause any harm to the human body;
(5) Simple production process, easy molding, and short production period;
(6) Stable physical and mechanical properties of the material, less affected by external influences;
(7) Simplification of the material production process and fast drying after molding to speed up the model testing process [16,31].

**Figure 1:**
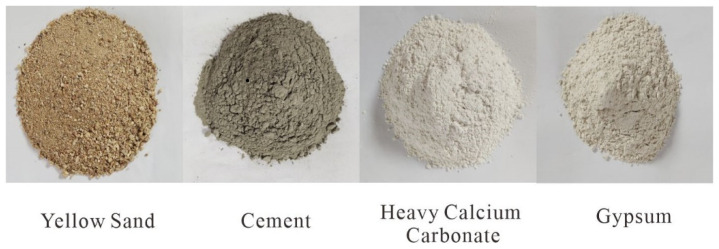
Similar material raw materials.

Aiming at the poor mechanical properties of coal system rocks, most of which are characterized by plasticity and elasticity, and a small part of which are accompanied by brittleness [32], and combined with the factor of striving for simple raw materials in this experiment, the following four common materials were selected as raw materials for similar materials: yellow sand (earthy yellow, density 1.69 g/cm^3^), heavy calcium carbonate (white, density 1.41 g/cm^3^), composite silicate cement (gray-black, density 1.26 g/cm^3^), and high-strength gypsum powder (off-white, density 1.17 g/cm^3^) (Figure 1). Among these, yellow sand is the coarse aggregate, heavy calcium carbonate is the fine aggregate, and the composite silicate cement and high-strength gypsum are the binders. Tap water was used as the mixing water without any additives and auxiliary materials.

### 2.2. Orthogonal Design Scheme

#### 2.2.1. Factors and Level Setting

From the perspective of solid materials, by controlling the mass ratio of aggregate to binder and the contents of cement and gypsum in the binder, and taking advantage of the differing strength properties of cement and gypsum after solidification, it is possible to induce a certain range of variations in the mechanical properties of similar materials.

Heavy calcium carbonate within the aggregate serves as a fine-grained material, effectively occupying the voids between the yellow sand particles and optimizing the gradation of the aggregate; in terms of mixing water, the degree of the hydration reaction of cement and gypsum as a binder material can be changed by varying the mass ratio of mixing water to solid material.

Taking into account the specific problem in this study, four variables were identified as control factors based on an orthogonal experimental design: A, aggregate–binder ratio (mass ratio of aggregate to binder); B, cement–gypsum ratio (mass ratio of cement to gypsum); C, calcium carbonate content (mass ratio of heavy calcium carbonate to aggregate); and D, moisture content (ratio of mixing water to solids). Each of these factors was assigned five levels. The aggregate–binder ratio was changed sequentially from 4:6 to 8:2; the calcium carbonate content was increased to 30% in steps of 5%, starting from 10%; the cement–gypsum ratio was changed sequentially from 3:7 to 7:3; and the moisture content was increased to 18% in increments of 2% starting from 10%, as depicted in Table 1.

#### 2.2.2. Orthogonal Design of Proportioning Scheme

A 6-factor, 5-level orthogonal table L_25_(5^6^) was selected for the experiment, and the orthogonal design scheme was generated using SPSSAU software (https://spssau.com/index.html, accessed on 8 May 2023). As it was not possible to fill the orthogonal table completely under the 4-factor condition, the last two columns were used as blank columns, and the specific scheme is shown in Table 2.

## 3. Sample Production and Testing Procedure

### 3.1. Sample Production

According to the Standard for Test Methods of Engineering Rock Mass (GB/T 50266-2013) [33], it is necessary to make a standard cylinder specimen with a size of Φ50 mm × 100 mm to obtain the uniaxial compressive strength, a standard cylinder specimen with a size of Φ50 mm × 50 mm to obtain the tensile strength, and a standard square specimen with a size of 70.7 mm × 70.7 mm × 70.7 mm to obtain the cohesion and the internal friction angle.

In this test, for the same ratio, 3 cylindrical specimens and 6 square specimens need to be made; 25 groups of ratios are being tested, so a total of 225 specimens are needed.

#### 3.1.1. Preliminary Preparation

The preliminary treatment of experimental raw materials and preparation of molds and sample-making tools are required before producing specimens. Among the experimental raw materials, yellow sand contains a large amount of moisture; in order to avoid the impact of moisture on the experimental results, the sand needs to be dried to contain no moisture in advance and then sieved. The remaining three materials were finished loose powder with no need for preliminary preparation. Specimen molds for the Φ50 mm × 100 mm and Φ50 mm × 50 mm standard cylinders were made of ABS (Acrylonitrile Butadiene Styrene plastic) material with removable molds, and the 70.7 mm × 70.7 mm × 70.7 mm standard square specimens were produced using triple molds. In addition, in the process of specimen production, we also needed to use the following equipment: a rubber hammer, compactor, geotechnical knife, electronic scales, trowel knife, plastic wrap, soft brush, petroleum jelly, and so on.

#### 3.1.2. Sample Production Process

Specimen production was carried out in groups, with one ratio per group. At the same time, in order to minimize human error in the process of operation, the production of samples was performed in a random order. The specific steps are as follows.

(1) Mold preparation: After cleaning the molds thoroughly, ensure that the surface moisture is wiped away (in the case of cylindrical molds, disassemble them for cleaning and reassemble after washing). Use a soft-bristle brush to evenly apply a layer of petroleum jelly to the inner walls of the molds. While applying, pay attention to potential omissions at the mold’s corners;
(2) Material preparation: According to the ratios provided in Table 2, calculate the quantities of yellow sand, calcium carbonate, cement, gypsum, and mixing water required for each group’s formulation. Weigh out the specified amounts and set them aside for later use;
(3) Material mixing: Due to the relatively small quantities of raw materials for each formulation, manual mixing is employed. To minimize errors in moisture content caused by water absorption from the ground, choose a non-absorbent surface for mixing whenever possible. After combining the materials mentioned in step (2), conduct a dry mix. Once the materials are uniformly mixed, add the mixing water all at once and continue stirring until a uniform mixture is achieved;
(4) Production of samples: Fill the mold with the mixed materials while compacting manually to prevent layering and ensure uniform filling. During this process, consider two aspects: ① For specimens of the same size, they should be produced by the same individual to minimize errors resulting from differences in compaction force. ② During the compaction process, pay attention to the condition of the seam on cylindrical molds. If excessive force causes the mold to bulge and deform, it is necessary to remake the specimen;
(5) Specimen smoothing. The surface of the specimen is smoothed with a geotechnical knife to keep the dimensions within the tolerance allowed;
(6) Specimen maintenance. In a conservation environment with room-temperature conditions, cover the surface of freshly prepared specimens with plastic wrap to minimize moisture loss; after two days of resting, remove the cling film and remove the mold (the original plan was to remove the mold after two hours of maintenance; however, in the pre-testing process, we found that the strength of the material in two hours did not meet the requirements and could not be normally demolded); if the specimen is damaged by the demolding process, it needs to be re-produced. Continue to condition the specimen for five days after demoulding for a total conditioning period of seven days;
(7) Specimen numbering. Number each set of specimens from 1 to 25 in accordance with the proportion of specimens.

### 3.2. The Testing Process

**Figure 2:**
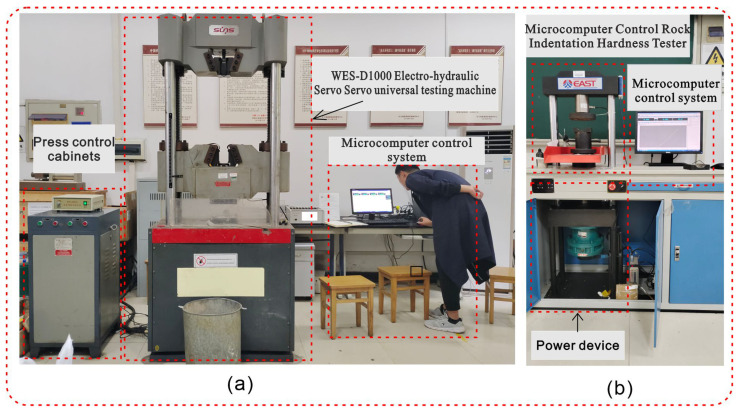
Main test equipment for mechanical property tests. ((**a**) is WES-D1000 electro-hydraulic servo servo universal testing machine, and (**b**) is microcomputer-controlled rock indentation hardness testing machine).

The mechanical testing of materials requires the use of equipment, such as the “WES-D1000 electro-hydraulic servo servo universal testing machine” (Sanshi Zongheng Technology Company Limited, Shenzhen, China) (Figure 2a) and “microcomputer-controlled rock indentation hardness testing machine” (Jinan Dongfang Testing Instrument Company Limited, Jinan, China) (Figure 2b).

**Figure 3:**
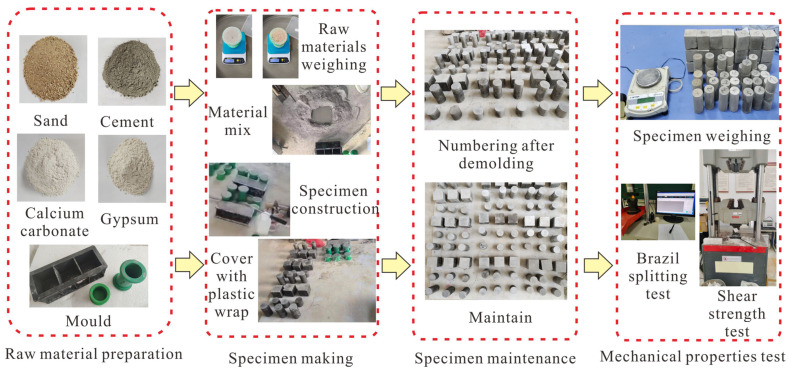
Similar material fabrication and mechanical property testing process.

The well-maintained specimens were tested indoors, and each specimen was weighed with an electronic balance before the test to obtain its quality parameters. Then, a “microcomputer-controlled rock indentation hardness tester” was used to conduct the “Brazilian split” test on the standard cylindrical specimen of Φ50 mm × 50 mm at a loading speed of 0.02 KN/s to indirectly obtain the tensile strength parameters of the material. We employed the WES-D1000 electro-hydraulic servo universal testing machine to test the standard cylindrical specimen measuring Φ50 mm × 100 mm and the standard square specimen measuring 70.7 mm × 70.7 mm × 70.7 mm. The testing was conducted at a loading rate of 2 KN/s to obtain the material’s uniaxial compressive strength parameters and shear strength parameters. The shear strength parameters were obtained by using the “shearing method with variable angle plates”, and the angles of the plate were selected to be 40°, 50°, and 60°, respectively. The process for preparing similar material test specimens and conducting mechanical property tests can be found in Figure 3.

## 4. Test Results

### 4.1. Overall Analysis of Test Results

The results of these tests were calculated according to the relevant formulas [33] to obtain the density, uniaxial compressive strength, tensile strength, cohesion, internal friction angle, and other physico-mechanical property indexes of the materials under different ratios, and the specific results are shown in Table 3.

**Figure 4:**
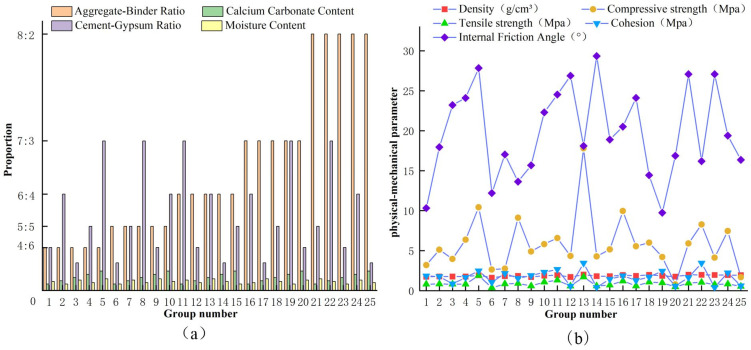
Trends of similar material proportioning and mechanical parameter changes. ((**a**) is the material ration, (**b**) is the material mechanical parameters).

The formulations of the 25 groups of materials and their mechanical parameters were visualized to gain an intuitive feel of the trends of the physical and mechanical properties of the materials with each factor, as shown in Figure 4.

Overall, the distribution of each physical–mechanical parameter of the similar materials ranged as follows: density, 1.63–2.01 g/cm^3^; uniaxial compressive strength, 0.78–17.81 MPa; tensile strength, 0.36–1.89 MPa; cohesion, 0.439–3.45 MPa; and internal friction angle, 9.74°–31.44°. There was a wide range of variation in the properties of similar materials.

### 4.2. Rock Types That Can Be Modeled by Similar Materials

According to the method of magnitude analysis in the similarity principle of the model test, the conventional mechanical parameters of the similar material and conventional mechanical parameters of the original rock need to satisfy the following equations [34,35]:

(1) $$\left\{\begin{array}{c} C_{\varepsilon }=C_{\mu }=C_{\varphi }=1\\ C_{\sigma }=C_{l}C_{\gamma }\\ C_{E}=C_{l}C_{\gamma }\\ C_{g}=C_{l}C_{v}^{2}\\ C_{t}=C_{v}^{-1}\\ C_{c}=C_{l}C_{\gamma } \end{array}\right.$$

In the equation, *C* represents the similarity ratio; subscript *ε* stands for strain; *μ* denotes Poisson’s ratio; *φ* signifies friction angle; σ represents stress; *l* signifies geometric dimensions; *γ* pertains to gravity; *E* stands for elastic modulus; *g* denotes gravitational acceleration; *t* corresponds to time; *v* represents volume; and *c* stands for cohesive strength.

From the equation, it can be observed that, for the dimensionless physical variable φ, similar materials should remain consistent with the original rock. Dimensional quantities, such as gravity and stress, can be controlled in model experiments by adjusting the similarity ratio scale to achieve the desired parameters.

Therefore, the internal friction angles of similar materials prepared in this test were compared with those of common rocks without considering the similar scale corresponding to specific engineering cases (Table 4). It can be found that the range of variation in the internal friction angle of the similar materials intersected with the range of variation of the internal friction angles of the primary rocks, such as main coking coal, anthracite, shale, schist, millimetalite, marl. Hence, under a certain similarity scale, the similar materials prepared in this test meet the simulation needs of main coking coal, anthracite, shale, marl, and other coal-system rocky protoliths in the similarity modeling test.

## 5. Sensitivity Analysis of Factors

The results of orthogonal tests are usually analyzed by means of range analysis and analysis of variance (ANOVA) [39]. For extreme variance analysis, in general, the effect of changing the level of each factor on the test results is different, and the magnitude of the extreme variance reflects the extent to which the factor affects the results of the test at different level changes. The larger the extreme variance, the greater the effect of the different levels under that factor on the test results (the more sensitive the test results are to the factor), and the factor with the largest extreme variance is the most dominant factor.

ANOVA provides a precise estimate of the significance of the effect of each factor on the results of the test based on the analysis of extreme variance, making the results more credible. Based on the test results, the following sensitivity analyses of the effects of the factors on the physical and mechanical properties of the materials were carried out by means of extreme variance analysis and ANOVA.

### 5.1. Range Analysis

#### 5.1.1. Analyses of Density Sensitivity

A sensitivity analysis was conducted on the factors influencing the material density within the orthogonal experimental results, and the results of the analysis are shown in Table 5. We observed that the ranges for the different factors (A, B, C, and D) were 0.19, 0.09, 0.14, and 0.09, respectively. It can be seen that the aggregate–binder ratio had the largest range of 0.19, followed by the cement–gypsum ratio, and finally the calcium carbonate content and moisture content. The sensitivity of each factor to density was, in descending order: aggregate–binder ratio > cement–gypsum ratio > calcium carbonate content = moisture content. This indicates that the different levels of the aggregate–binder ratio had a significant effect on the density of the material, with the aggregate–binder ratio playing a major role in controlling the density. In fact, within the design of the four factors, the raw material’s aggregate density exceeded 1.4 g/cm^3^, while the binder’s density was below 1.3 g/cm^3^. The aggregate’s density was indeed higher than that of the binder, aligning well with the test results.

**Figure 5:**
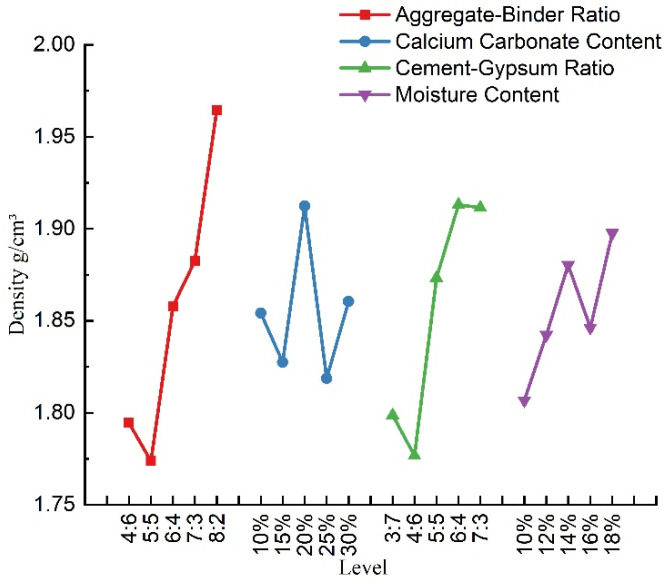
Density sensitivity analysis.

According to Table 5, a visual analysis of the effect of each factor on the density of the material was conducted, as shown in Figure 5. It can be seen that, in the range of this test ratio, the density of similar materials with an increase in the aggregate–binder ratio showed an increasing trend; with an increase in the calcium carbonate content, it showed an oscillating change; with an increase in the cement–gypsum ratio, it first decreased and then increased; with an increase in the moisture content, it showed the trend of firstly increasing, then decreasing, and then increasing; the changes in the moisture content and the content of calcium carbonate did not have a significant effect on the density. In practice, the density of a material can be increased by increasing the aggregate–binder ratio when formulating the material.

#### 5.1.2. Sensitivity Analysis of Uniaxial Compressive Strength

A sensitivity analysis was performed on the factors affecting the uniaxial compressive strength of the material within the orthogonal experimental results. The outcomes are presented in Table 6. We observed that the range values of the different factors (A, B, C, and D) were 2.59, 3.43, 5.76, and 4.67, respectively, from which it can be seen that the cement–gypsum ratio had the highest extreme value of 5.76, followed by the moisture content, then the calcium carbonate content, and lastly the aggregate–binder ratio. The sensitivities of the factors to the uniaxial compressive strength in descending order were cement–gypsum ratio > moisture content > calcium carbonate content > aggregate–binder ratio. This indicates that different levels of the cement–gypsum ratio had a significant effect on the uniaxial compressive strength of the material. The primary control on uniaxial compressive strength was the cement–gypsum ratio, while the rest of the factors played a secondary role in the compressive strength.

**Figure 6:**
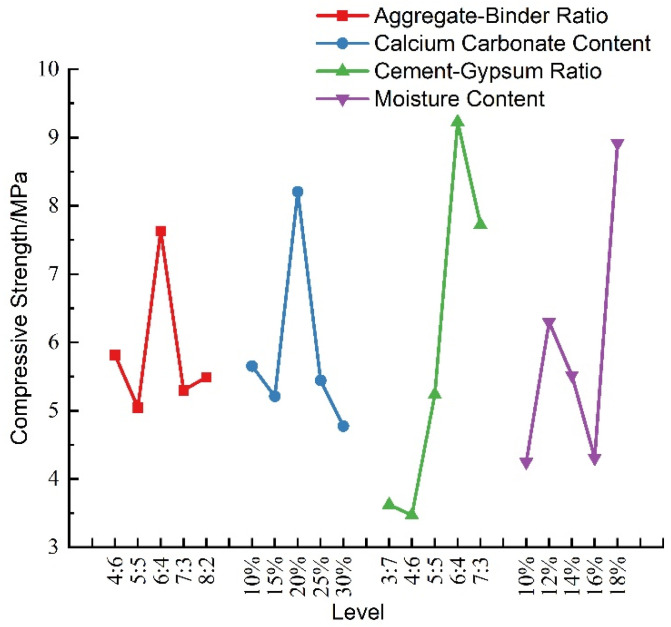
Sensitivity analysis of uniaxial compressive strength.

The visual analysis of the effect of each factor on the compressive strength of the materials was plotted according to Table 6, as shown in Figure 6. It can be seen that, within the range of ratios of this test, the uniaxial compressive strength of similar materials varied irregularly with the increase in the aggregate–binder ratio and the increase in the calcium carbonate content. The compressive strength first increased and then decreased with the increase in the cement–gypsum ratio. The compressive strength of the material was maximum when the cement–gypsum ratio was 6:4, indicating that, at this level, it was able to withstand the maximum compressive stress as the ratio of cement to gypsum was optimal. The compressive strength with the increase in moisture content showed a trend of first increasing, then decreasing, and then increasing, indicating that the moisture content had a certain effect on the compressive strength of the material; the changes in the aggregate–binder ratio and calcium carbonate content did not have a significant effect on the uniaxial compressive strength. Therefore, the magnitude of compressive strength can be adjusted in practice by changing the ratio of cement to gypsum in the material.

#### 5.1.3. Sensitivity Analysis of Tensile Strength

The results of the orthogonal tests were analyzed in terms of extreme variance for each factor affecting the tensile strength of the material, and the results are shown in Table 7. We observed that the ranges of different factors (A, B, C, and D) were 0.28, 0.29, 0.65, and 0.42, respectively. It can be seen that the greatest range was associated with the cement–gypsum ratio, reaching 0.65, followed by the moisture content. The calcium carbonate content and aggregate–binder ratio exhibited the smallest ranges. Therefore, in terms of sensitivity to tensile strength, the factors can be ranked from the most influential to the least influential as follows: cement–gypsum ratio > moisture content > calcium carbonate content > aggregate–binder ratio. This indicates that different levels of the cement–gypsum ratio had a greater effect on the tensile strength of the material. It is cement–gypsum that had the main control over the tensile strength, and the rest of the factors played a secondary role.

**Figure 7:**
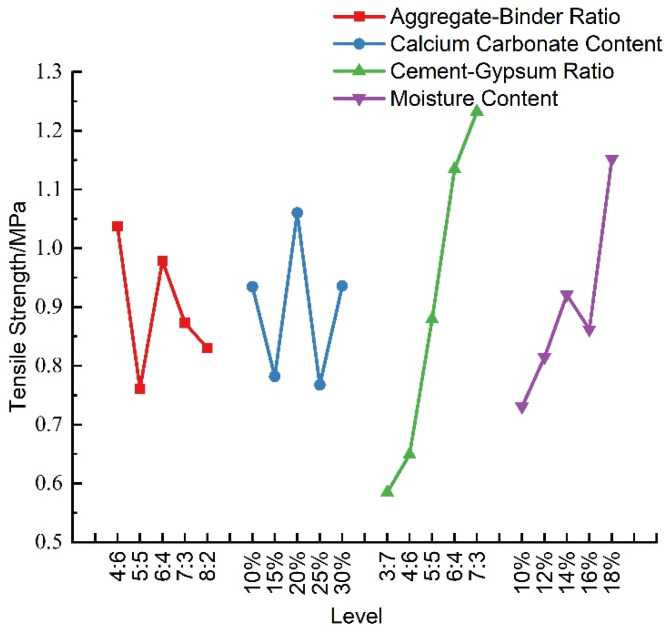
Sensitivity analysis of tensile strength.

According to Table 7, a visual analysis of the effect of each factor on the tensile strength of the material was performed, as shown in Figure 7. It can be observed that the tensile strength of the similar materials exhibited irregular fluctuations with the enlargement of the aggregate–binder ratio and the augmentation of the calcium carbonate content. As the cement–gypsum ratio increased, the tensile strength increased almost linearly, which indicates that the content of cement played an important role in the tensile strength: the more cement, the larger the cement–gypsum ratio, and the stronger the tensile strength. Therefore, in practical applications, the size of the tensile strength of the material can be changed by adjusting the cement–gypsum ratio. In addition, the increase in the moisture content caused the tensile strength to show a tendency to increase, then decrease, and then increase, while the change in the aggregate–binder ratio and calcium carbonate content did not have a significant effect on the tensile strength.

#### 5.1.4. Sensitivity Analysis of Cohesive Strength

An extreme variance analysis of the factors that impacted the material’s cohesive strength within the orthogonal experimental results was performed, as shown in Table 8. From the data, it can be observed that the range among different factors (A, B, C, and D) varied, with values of 0.22, 0.37, 1.67, and 0.90, respectively. This analysis reveals that the cement–gypsum ratio exhibited the greatest range, reaching a value of 1.67, followed by the moisture content. Meanwhile, the calcium carbonate content and aggregate–binder ratio showed smaller ranges. Concerning their impact on cohesion, the factors can be ranked in descending order of influence as follows: cement–gypsum ratio > moisture content > calcium carbonate content > aggregate–binder ratio. This observation underscores the significant impact of different levels of cement–gypsum ratio on the material’s cohesion. The cement–gypsum ratio primarily influenced cohesion and played a pivotal role in determining it.

**Figure 8:**
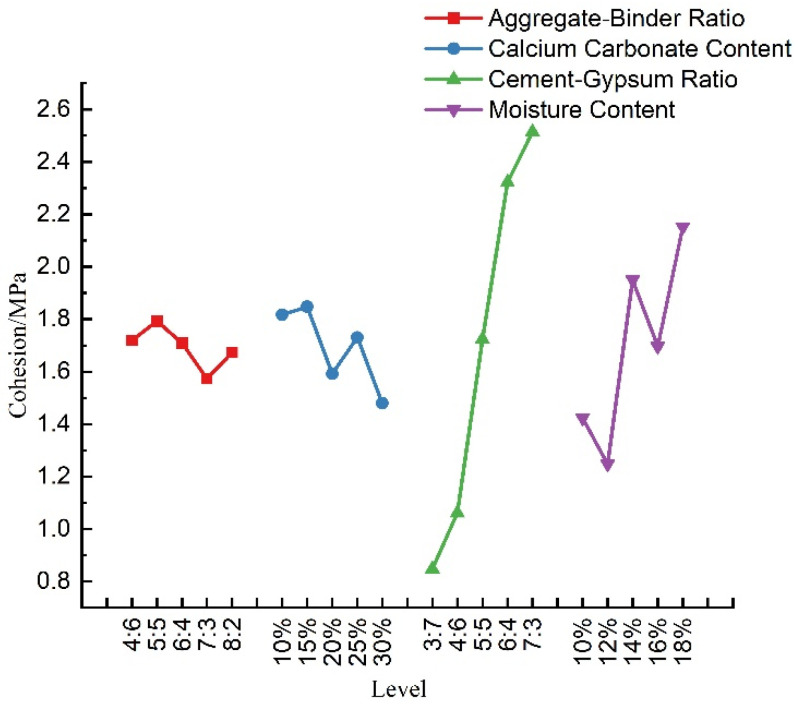
Sensitivity analysis of cohesion.

According to Table 8, a visual analysis of the effect of each factor on the cohesion of the material was conducted, as shown in Figure 8. It can be seen that, in the range of this test ratio, the cohesion of similar materials with an increase in the aggregate–binder ratio and calcium carbonate content showed a decreasing trend, while the cohesion with an increase in the cement–gypsum ratio showed an obvious linear increasing trend. In theory, cohesion is related to the bonding strength between particles within the material. The higher the content of cement, which is a strong binding material, the better the bonding between particles, resulting in an increase in the material’s cohesion. This aligns with our experimental findings. Therefore, the cohesion of the material can be increased by increasing the cement–gypsum ratio in practical applications. In addition, the cohesive force also showed an increasing trend with the increase in moisture content, while the changes in the aggregate–binder ratio and calcium carbonate content did not have a significant effect on the cohesive force.

#### 5.1.5. Sensitivity Analysis of Internal Friction Angle

The orthogonal test results of the factors affecting the angle of internal friction of the material were analyzed in terms of extreme deviation, and the results are shown in Table 9. We observed that the ranges of different factors (A, B, C, and D) were 7.81, 1.52, 3.08, and 5.39, respectively. Notably, the aggregate–binder ratio exhibited the largest range, reaching 7.81, followed by the moisture content. In contrast, the cement–gypsum ratio and calcium carbonate content showed smaller ranges. When considering sensitivity to the internal friction angle, the factors can be ranked from the most influential to the least influential as follows: aggregate–binder ratio > moisture content > cement–gypsum ratio > calcium carbonate content. This indicates that the different levels of the aggregate–binder ratio had a significant impact on the material’s internal friction angle. The aggregate–binder ratio was the primary controlling factor for the internal friction angle, while the other factors played secondary roles. This underscores the primary role of the aggregate–binder ratio in controlling the internal friction angle.

**Figure 9:**
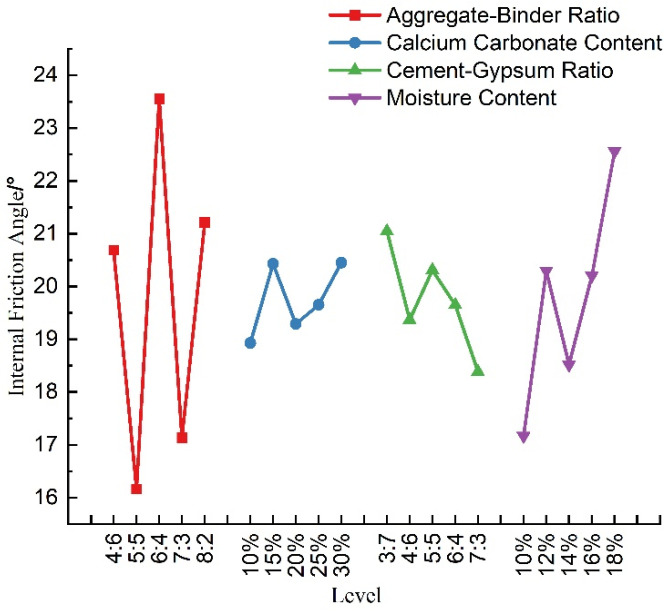
Sensitivity analysis of internal friction angle.

A visual analysis of the effect of each factor on the angle of internal friction of the material was performed according to Table 9 and is shown in Figure 9. From this, it can be observed that, within the range of compositions tested in this experiment, the internal friction angle of the simulant material exhibited irregular variations as the bone glue ratio increased. The maximum internal friction angle was achieved when the bone glue ratio was 6:4, which may be related to the particle state within the material. The internal friction angle showed a gradual upward trend with an increase in calcium carbonate content; it demonstrated a decreasing trend with an increase in the cement–gypsum ratio, and it displayed an initial increase, followed by a decrease, and then an increase with the increase in the moisture content. Among these, alterations in the calcium carbonate content and the cement–gypsum ratio had relatively inconspicuous effects on the internal friction angle. In fact, the angle of internal friction reflected the degree of occlusion between particles, and the more closely the particles were embedded in each other, the greater the angle of internal friction. The aggregate in the material was mostly coarse particles, and the binder was composed of fine particles. When the aggregate–binder ratio changed, the filling of the pores between the fine particles on the coarse particles and the particle gradation changed, which, in turn, affected the tightness of the bond between the particles, which is reflected in the change in the angle of internal friction. Consequently, in practical applications, adjusting the ratio between the aggregate and binder can effectively modify the internal friction angle of similar materials.

### 5.2. Analysis of Variance (ANOVA)

Compared with range analysis, ANOVA can identify the factors that have a significant impact on experimental results, reveal interactions between various factors, and determine the optimal levels of influential factors [40]. In ANOVA, the *p*-value returned using the F-distribution can be used to assess whether there is a statistically significant difference between the means of different groups according to the test of significance. In general, *p* > 0.05 was considered insignificant (—), 0.01 < *p* < 0.05 was considered significant (*), and *p* < 0.01 was considered highly significant (**). The following is a one-way ANOVA for the four factors affecting the nature of the material based on the results of the orthogonal test. The *p*-value under each factor was calculated using Excel software (Office 19), and the significance level of the effect of each factor was discussed. The results are shown in Table 10.

In the density ANOVA, factor A (aggregate–binder ratio) showed a highly significant effect (*p* = 0.004), factor C (cement–gypsum ratio) showed a significant effect (*p* = 0.012), and the rest of the factors caused a non-significant effect. Thus, the choice of the aggregate–binder ratio is a critical parameter for density when modeling similar materials of coal-based rock. For compressive strength, factor C (cement–gypsum ratio) showed a highly significant effect (*p* = 0.003), and factor D (moisture content) showed a significant effect (*p* = 0.018). This implies that the adjustment of the cement–gypsum ratio and moisture content is essential to improving the compressive strength of the materials. The ANOVA results of tensile strength showed that factor C (cement–gypsum ratio) had a highly significant effect on tensile strength (*p* = 0.005), while other factors did not show significance. Therefore, the adjustment of the cement–gypsum ratio may be decisive in improving the tensile strength when modeling similar materials to coal system rock. The results of the analysis of cohesion showed that only factor C (cement–gypsum ratio) had a significant effect on cohesion (*p* = 0.012), which implies that the cement–gypsum ratio is the primary cause of material cohesion in the test. Finally, none of the four factors showed a significant effect in the ANOVA of the internal friction angle. This suggests that the selection of these factors may not be critical in influencing the internal friction angle when modeling similar materials to coal system rock.

In summary, the results of the above range analysis and ANOVA provide a reference for selecting suitable raw materials for the preparation of coal system rocky similar materials. In practical applications, the results of these analyses can be used to optimize the formulations of coal system rock-like materials to meet the needs of different model tests.

## 6. Conclusions

The physical and mechanical parameters of the coal system rocky similar materials formulated with yellow sand, heavy calcium carbonate, cement, and gypsum as raw materials are widely distributed, which can meet the simulation needs of different types of coal-based rocks, such as main coking coal, anthracite, shale, marl, etc., in similarity modeling tests. The purpose of simulating a variety of coal beds by changing the ratio of raw materials was realized, which solves the problem of complex raw materials or the single simulation results of the previous work.

The sensitivity of the factors to the physico-mechanical parameters of the materials at different ratios was analyzed by using range analysis combined with visual diagrams. Our findings reveal that the material’s density is predominantly influenced by the aggregate–binder ratio, with an increase in this ratio resulting in higher material density. Compressive strength is primarily governed by the cement–gypsum ratio, reaching its peak when the ratio was set at 6:4. The tensile strength was primarily controlled by the cement–gypsum ratio, and an increase in this ratio significantly increased the material’s tensile strength. Cohesion was chiefly regulated by the cement–gypsum ratio, and an increase in the cement–gypsum ratio led to a corresponding increase in the material’s cohesion. The internal friction angle was greatly affected by the aggregate–binder ratio.

An analysis of the significance levels of various factors affecting the material’s physical mechanical parameters was conducted using ANOVA. The results indicated that the influence of the aggregate–binder ratio on density, as well as the influence of the cement–gypsum ratio on compressive strength and tensile strength, was highly significant. The cement–gypsum ratio significantly affected density and cohesion, while moisture content significantly influenced compressive strength. The remaining factors were not significant. Furthermore, changes in moisture content during the production of similar materials can lead to large changes in parameters such as the tensile strength, compressive strength, and cohesion of the material. Therefore, the amount of moisture added during the mixing process for preparing similar materials should be adjusted according to the specific conditions.

The test shows that the pathway of using simple raw materials to formulate coal system rocky similar materials is feasible. The proportioning scheme and test outcomes presented in this paper can serve as valuable references for the formulation of similar materials in related physical modeling tests. However, the physico-mechanical properties of the similar materials under triaxial circumferential pressure were not measured, and further research is needed.

## Figures and Tables

**Table 1:** Orthogonal design levels of similar materials.

Levels	Aggregate–Binder Ratio	Calcium Carbonate Content	Cement–Gypsum Ratio	Moisture Content
1	4:6	10%	3:7	10%
2	5:5	15%	4:6	12%
3	6:4	20%	5:5	14%
4	7:3	25%	6:4	16%
5	8:2	30%	7:3	18%

**Table 2:** Similar material proportioning scheme.

Number	A (Aggregate–Binder Ratio)	B (Calcium Carbonate Content)	C (Cement–Gypsum Ratio)	D (Moisture Content)	E	F
1	4:6	10%	4:6	14%	4	2
2	4:6	15%	6:4	10%	3	3
3	4:6	20%	3:7	16%	2	4
4	4:6	25%	5:5	12%	1	5
5	4:6	30%	7:3	18%	5	1
6	5:5	10%	3:7	10%	1	1
7	5:5	15%	5:5	16%	5	2
8	5:5	20%	7:3	12%	4	3
9	5:5	25%	4:6	18%	3	4
10	5:5	30%	6:4	14%	2	5
11	6:4	10%	7:3	16%	3	5
12	6:4	15%	4:6	12%	2	1
13	6:4	20%	6:4	18%	1	2
14	6:4	25%	3:7	14%	5	3
15	6:4	30%	5:5	10%	4	4
16	7:3	10%	6:4	12%	5	4
17	7:3	15%	3:7	18%	4	5
18	7:3	20%	5:5	14%	3	1
19	7:3	25%	7:3	10%	2	2
20	7:3	30%	4:6	16%	1	3
21	8:2	10%	5:5	18%	2	3
22	8:2	15%	7:3	14%	1	4
23	8:2	20%	4:6	10%	5	5
24	8:2	25%	6:4	16%	4	1
25	8:2	30%	3:7	12%	3	2

Note: The above ratios are all mass ratios.

**Table 3:** Mechanical parameters of similar materials.

Number	Density/(g/cm^3^)	Uniaxial Compressive Strength/MPa	Tensile Strength/MPa	Cohesion C/MPa	Internal Friction Angle φ/°
1	1.75	3.21	0.82	1.85	10.33
2	1.77	5.11	0.83	1.76	17.95
3	1.76	3.97	0.83	0.85	23.21
4	1.76	6.38	0.81	1.65	24.11
5	1.93	10.42	1.89	2.48	27.84
6	1.63	2.63	0.36	1.07	12.20
7	1.82	2.76	0.85	2.16	17.03
8	1.84	9.13	0.92	1.54	13.63
9	1.71	4.90	0.60	1.89	15.67
10	1.87	5.82	1.07	2.30	22.30
11	1.94	6.58	1.31	2.66	24.53
12	1.71	4.34	0.58	0.58	26.89
13	2.01	17.81	1.71	3.45	18.11
14	1.82	4.28	0.59	0.39	31.44
15	1.81	5.16	0.70	1.43	18.89
16	1.96	9.95	1.22	1.85	20.51
17	1.85	5.56	0.60	1.28	24.11
18	1.98	6.01	1.08	1.72	14.44
19	1.86	4.20	0.99	2.44	9.74
20	1.76	0.78	0.48	0.57	16.87
21	1.99	5.90	0.95	1.65	27.08
22	1.98	8.28	1.04	3.45	16.18
23	1.96	4.13	0.77	0.41	27.08
24	1.95	7.45	0.84	2.24	19.39
25	1.94	1.69	0.54	0.61	16.36

**Table 4:** Partial rock mechanical strength parameters [36,37,38].

Rock Type	Density/ (g/cm^3^)	Uniaxial Compressive Strength/MPa	Cohesion/MPa	Internal Friction Angle/(°)
Main Coking Coal	1.2–1.7	4.9–49	0.98–9.81	16–40
Anthracite	1.2–1.7	9.81–15.7	1.96–3.92	28–35
Chimneystone, Schist	2.5–3.3	10–100	1–20	26–65
Shale	2.1–2.8	4.5–78.5	6.63–20	11–60
Marl	2.3–2.5	3.5–20	200–700	9–65
Similar materials	1.63–2.01	0.78–17.81	0.39–3.45	9.74–31.44

**Table 5:** Range analysis of density.

Level Average	Aggregate–Binder Ratio	Calcium Carbonate Content/%	Cement–Gypsum Ratio	Moisture Content/%
k1	1.79	1.85	1.78	1.88
k2	1.77	1.83	1.91	1.81
k3	1.86	1.91	1.80	1.85
k4	1.88	1.82	1.87	1.84
k5	1.96	1.86	1.91	1.90
Range	0.19	0.09	0.14	0.09

**Table 6:** Range analysis of uniaxial compressive strength.

Level Average	Aggregate–Binder Ratio	Calcium Carbonate Content/%	Cement–Gypsum Ratio	Moisture Content/%
k1	5.82	5.65	3.47	5.52
k2	5.05	5.21	9.23	4.25
k3	7.63	8.21	3.62	4.31
k4	5.30	5.44	5.24	6.30
k5	5.49	4.77	7.72	8.92
Range	2.59	3.43	5.76	4.67

**Table 7:** Range analysis of tensile strength.

Level Average	Aggregate–Binder Ratio	Calcium Carbonate Content/%	Cement–Gypsum Ratio	Moisture Content/%
k1	1.04	0.93	0.65	0.92
k2	0.76	0.78	1.13	0.73
k3	0.98	1.06	0.58	0.86
k4	0.87	0.77	0.88	0.81
k5	0.83	0.94	1.23	1.15
Range	0.28	0.29	0.65	0.42

**Table 8:** Range analysis of cohesion.

Level Average	Aggregate–Binder Ratio	Calcium Carbonate Content/%	Cement–Gypsum Ratio	Moisture Content/%
k1	1.72	1.82	1.06	1.94
k2	1.79	1.85	2.32	1.42
k3	1.70	1.59	0.84	1.70
k4	1.57	1.72	1.72	1.25
k5	1.67	1.48	2.51	2.15
Range	0.22	0.37	1.67	0.90

**Table 9:** Range analysis of internal friction angle.

Level Average	Aggregate–Binder Ratio	Calcium Carbonate Content/%	Cement–Gypsum Ratio	Moisture Content/%
k1	20.69	18.93	19.37	18.94
k2	16.16	20.43	19.65	17.17
k3	23.97	19.29	21.46	20.21
k4	17.14	20.07	20.31	20.30
k5	21.22	20.45	18.39	22.56
Range	7.81	1.52	3.08	5.39

**Table 10:** ANOVA of influencing factors on mechanical parameters of similar materials.

Factor	Aggregate–Binder Ratio	Calcium Carbonate Content/%	Cement–Gypsum Ratio	Moisture Content/%	Error
Density	SS_A_:0.115	SS_B_:0.027	SS_C_:0.081	SS_D_:0.025	SSe:0.025
	df_A_:4	df_B_:4	df_C_:4	df_D_:4	dfe:8
	MS_A_:0.029	MS_B_:0.007	MS_C_:0.02	MS_D_:0.006	MSe:0.003
	F_A_:9.406	F_B_:2.202	F_C_:6.619	F_D_:2.054	
	P_A_:0.004	P_B_:0.159	P_C_:0.012	P_D_:0.179	
	**	—	*	—	
Compressive	SS_A_:21.275	SS_B_:36.654	SS_C_:129.529	SS_D_:73.324	SSe:25.87
	df_A_:4	df_B_:4	df_C_:4	df_D_:4	dfe:8
	MS_A_:5.319	MS_B_:9.163	MS_C_:32.382	MS_D_:18.331	MSe:3.234
	F_A_:1.645	F_B_:2.834	F_C_:10.014	F_D_:5.669	
	P_A_:0.254	P_B_:0.098	P_C_:0.003	P_D_:0.018	
	—	—	**	*	
Tensile Strength	SS_A_:0.249	SS_B_:0.298	SS_C_:1.64	SS_D_:0.505	SSe:0.377
	df_A_:4	df_B_:4	df_C_:4	df_D_:4	dfe:8
	MS_A_:0.062	MS_B_:0.074	MS_C_:0.41	MS_D_:0.126	MSe:0.047
	F_A_:1.319	F_B_:1.579	F_C_:8.703	F_D_:2.681	
	P_A_:0.341	P_B_:0.27	P_C_:0.005	P_D_:0.11	
	—	—	**	—	
Cohesion	SS_A_:0.127	SS_B_:0.48	SS_C_:10.972	SS_D_:2.723	SSe:3.285
	df_A_:4	df_B_:4	df_C_:4	df_D_:4	dfe:8
	MS_A_:0.032	MS_B_:0.12	MS_C_:2.743	MS_D_:0.681	MSe:0.411
	F_A_:0.076	F_B_:0.288	F_C_:6.585	F_D_:1.634	
	P_A_:0.987	P_B_:0.878	P_C_:0.012	P_D_:0.249	
	—	—	*	—	
Internal Friction	SS_A_:202.604	SS_B_:9.546	SS_C_:26.171	SS_D_:78.474	SSe:498.663
	df_A_:4	df_B_:4	df_C_:4	df_D_:4	dfe:8
	MS_A_:50.651	MS_B_:2.387	MS_C_:6.543	MS_D_:19.618	MSe:62.333
	F_A_:0.818	F_B_:0.038	F_C_:0.105	F_D_:0.315	
	P_A_:0.551	P_B_:0.997	P_C_:0.978	P_D_:0.86	
	—	—	—	—	

SS_A–e_: sum of squares for factor A–e. df_A–e_: Degree of freedom corresponding to SS_A–e._ MS_A–e_: Mean squares between groups for factor A–e. F_A–D_: F-test for factor A–D. P_A–D_: Significance value of factor A–D. **: Highly significant. —: Insignificant. *: Significant.

## Data Availability

Data are contained within the article.
